# Evolution of the Apicomplexan Sugar Transporter Gene Family Repertoire

**DOI:** 10.1155/2017/1707231

**Published:** 2017-05-07

**Authors:** Ousman Mahmud, Jessica C. Kissinger

**Affiliations:** ^1^Department of Genetics, University of Georgia, Athens, GA 30602, USA; ^2^Center for Tropical and Emerging Global Diseases, University of Georgia, Athens, GA 30602, USA; ^3^Institute of Bioinformatics, University of Georgia, Athens, GA 30602, USA

## Abstract

Apicomplexan protist parasites utilize host sugars transported into the parasite by sugar transporter proteins for use as an energy source. We performed a phylum-wide phylogenetic analysis of the apicomplexan sugar transporter repertoire. Phylogenetic analyses revealed six major subfamilies of apicomplexan sugar transporters. Transporters in one subfamily have undergone expansions in Piroplasma species and *Gregarina niphandrodes*, while other subfamilies are highly divergent and contain genes found in only one or two species. Analyses of the divergent apicomplexan subfamilies revealed their presence in ciliates, indicating their alveolate ancestry and subsequent loss in chromerids and many apicomplexans.

## 1. Introduction

The sugar transporter gene family is one of the 25 gene families that make up the major facilitator superfamily (MFS) [1]. Members of the MFS are found in all domains of life [1, 2]. Genes in the MFS encode transporter proteins that mediate movement of a wide range of substrates across membranes [2]. Based on transport mode, MFS transporters are divided into three main groups: uniporters which transport a single substrate; symporters which transport a substrate in association with a coupling ion; and antiporters which transport a substrate and a cosubstrate in opposite directions [3]. Sugar transporters mediate the import of sucrose, monosaccharides (such as glucose, mannose, and fructose), and polyols (such as mannitol, sorbitol, and galactinol) [4]. In a phylogenetic context, monosaccharide transporters are further divided into several subfamilies such as hexose, tonoplast/vacuolar, and inositol [4]. Sugar transporter proteins can be found localized to the plasma membrane and subcellular compartments [5].

The phylum Apicomplexa contains primarily obligate intracellular parasites, most notably species including *Plasmodium*, *Babesia*, *Theileria*, *Toxoplasma*, *Eimeria*, and *Cryptosporidium*. Apicomplexans are the causative agents of significant diseases of humans and animals like malaria, toxoplasmosis, and cryptosporidiosis, which affect millions. The most notorious of these diseases, malaria caused by *Plasmodium* spp., affected at least 214 million people and caused 438 thousand deaths in 2015 [6]. The antiquity of the apicomplexan phylum (last common ancestor is ~500 million years old [7]) coupled with the availability of numerous genome sequences allows for the examination of genome evolution in an ancient phylum [8]. Apicomplexans have reductive streamlined genomes, that range from ~8.5 to ~125 megabases, and correlate roughly with the number of protein-encoding genes (~3650 to ~8000) [9–11]. Given the streamlined nature of these eukaryotic genome sequences, analyses of gene gain and loss patterns will be highly informative with respect to our understanding of the biology and evolution of parasitism.

Apicomplexan parasites utilize host sugars, transported into the parasite via sugar transporter proteins, as a source of energy [12, 13]. Individual sugar transporter proteins have been previously characterized in various apicomplexans: *Babesia bovis* (BboHT1), *Plasmodium falciparum* (PfHT1), *Plasmodium knowlesi* (PkHT1), *Plasmodium yoelii* (PyHT1), *Plasmodium vivax* (PvHT), *Plasmodium berghei* (PbHT1), and *Toxoplasma gondii* (TgGT1, TgST1, TgST2, and TgST3) [12–16]. Studies of the *Toxoplasma gondii* sugar transporter, TgGT1, revealed its ability to transport several hexose sugars (glucose, mannose, fructose, and galactose) [13]. There are differences in the protein localization patterns of *T. gondii* sugar transporters. The protein products of TgGT1 and another *T. gondii* sugar transporter, TgST2, were shown to localize in the plasma membrane of tachyzoites. The protein products of the two other *T. gondii* sugar transporters, TgST1 and TgST3, were shown to localize to intracellular vesicles in tachyzoites [13]. The characterized sugar transporters of *Plasmodium* species have been shown to localize in the plasma membrane during the asexual intraerythrocytic parasite stages, and they transport glucose and fructose [12]. The *T. gondii* sugar transporter TgGT1—which is the ortholog of the characterized *Plasmodium falciparum* sugar transporter, PfHT1—is not essential for the survival of tachyzoites, while PfHT1 is essential for the survival of the asexual intraerythrocytic stages [12, 13, 15, 17]. These differences in the essentiality of sugar transporters in *T. gondii* and *Plasmodium* species likely reflect adaptations to diverse environments. The substrate affinities, life stage essentiality, and protein localization patterns of characterized apicomplexan sugar transporters suggest the presence of several distinct subfamilies in this phylum.

Little is known about the evolution and diversity of the sugar transporter gene family in the Apicomplexa. To gain greater insight into the evolution of the apicomplexan sugar transporter gene family, we analyzed the apicomplexan repertoire to infer the ancestral state of the gene family. We examined trends in the evolutionary expansion and contraction of this family and asked, when possible, if the trends can be correlated with the biology of the parasites. We combined the apicomplexan sugar transporter phylogeny with publicly available functional genomics data to examine the biology of gene family members. We found that the variable number of apicomplexan sugar transporters present is a result of the expansion of hexose-like transporters combined with differential retention of transporter family members in different apicomplexan lineages. We found six different phylogenetic subfamilies of apicomplexan sugar transporters. *Cryptosporidium* and *Plasmodium* species contain divergent sugar transporters. The available functional genomics data suggest that the divergent sugar transporters in *Plasmodium* species are sporozoite-specific.

## 2. Methods

### 2.1. In Silico Identification and Validation of Apicomplexan Sugar Transporters

Twenty-two species were utilized in this study including 20 apicomplexans representing five major lineages (Cryptosporidia, Coccidia, Piroplasmida, and *Plasmodium* spp., as well as the deep branching apicomplexan, *Gregarina niphandrodes*) (Supplementary Table 1 available online at https://doi.org/10.1155/2017/1707231). The chromerids, *Chromera velia* and *Vitrella brassicaformis*, were included as outgroups. The chromerids are the closest free-living relatives of the Apicomplexa [18]. Protein and genome sequences were downloaded from EuPathDB (Supplementary Table 1). An ortholog clustering approach was used to identify orthologous proteins. Annotated proteins were clustered into orthologs and paralogs using OrthoMCL [19]. Custom Unix and Perl scripts (available on request) were used to parse orthology datasets and identify gene copy number patterns. Presence and absence of orthologs was used to determine patterns of gene gain and loss.

Pfam [20] scans of the annotated proteins were performed to identify and confirm apicomplexan and outgroup sugar transporters' copy number. The HMMER tool [21] was used to generate a hidden Markov model (HMM) from the multiple sequence alignments of identified sugar transporters, and this new HMM was used to search for additional sugar transporters that may have been missed in the annotated proteins. Translated BLAST (tBLASTn) searches [22] were performed to identify sugar transporters that may not have been annotated. CAFE [23] was used to analyze expansions and contractions within the sugar transporter gene family across apicomplexans and chromerids. Parameters for all computational analyses are presented in Supplementary Table 2.

### 2.2. Phylogenetic Analyses

Maximum likelihood and Bayesian approaches were used for phylogenetic analyses. TCoffee was used for the initial multiple sequence alignment (MSA) [24]. The resulting MSA was visualized and edited manually using Jalview 2.8 [25]. The Whelan and Goldman (WAG) amino acid substitution model [26] was used to infer the phylogenetic tree using only confidently aligned regions of the MSA (Gamma parameter and proportion of invariable sites were estimated). WAG was determined to be the best-fitting model using Modeltest within MEGA 5.2 [27]. Support for the reliability of the estimated phylogenetic tree was assessed using the likelihood ratio test, bootstrap, and posterior probabilities. Tree construction and evaluation were performed using PhyML located at the phylogeny.fr webserver [28] and BEAST [29]. The tree was visualized using Figtree (http://tree.bio.ed.ac.uk/software/figtree/).

### 2.3. Prediction of Subcellular Localization

TargetP 1.1 and SignalP 4.1 were used to predict the presence of signal and transit peptides in the identified sugar transporter proteins [30]. We also searched the upstream sequences (at least 1000 base pairs or until a stop codon is encountered in all three frames) of identified sugar transporter for peptides that may not have been annotated. Protein sequences with both signal and transit peptides, only transit peptide, or only signal peptide are predicted to be in the apicoplast or mitochondria or are classified as secretory proteins, respectively. Parameters for all computational analyses are in Supplementary Table 2.

### 2.4. Analyses of Functional Genomics Data

Publicly available RNA and protein datasets were mined to profile expression of *P. falciparum* sugar transporters. All proteomic expression data were obtained from EuPathDB (release 28) [9]. The threshold for evidence of protein expression was set at five peptides.

## 3. Results and Discussion

### 3.1. Sugar Transporter Gene Family Number Varies in Apicomplexans

**Figure 1:**
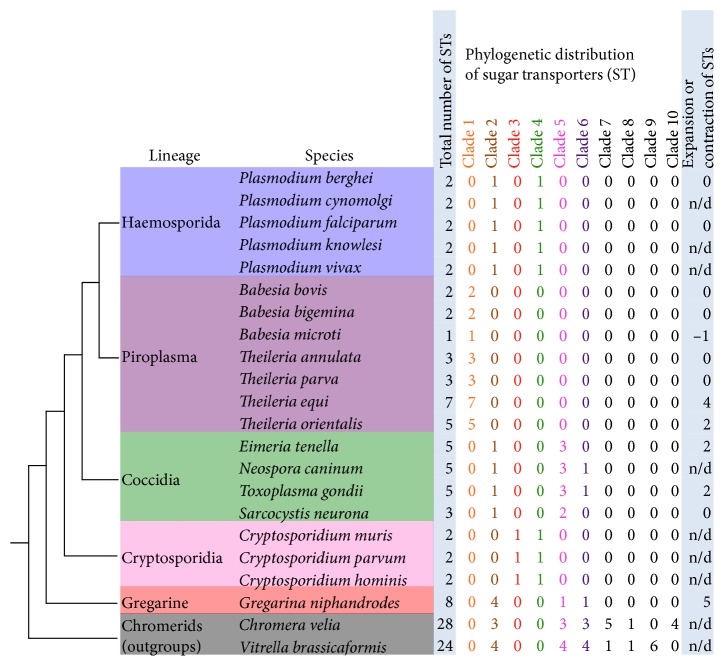
Distribution of apicomplexan sugar transporter (ST) gene family members. Left—cladogram shows relationships among apicomplexan lineages/species (Haemosporidia, Piroplasma, Coccidia, Cryptosporidia, and *Gregarina niphandrodes*) and their free-living outgroups: *Chromera velia* and *Vitrella brassicaformis* (Chromerids). Right—the first column lists the size of the sugar transporter gene family. The last column lists estimated expansion/contraction of sugar transporters in each species (see Supplementary Figure 1). Clades 2 and 5 have low bootstrap support in deeper branches (see Figure 2). n/d: not determined.

Gene gain and loss patterns in apicomplexans were identified using an orthology clustering approach [19]. Analysis of ortholog distribution revealed variation in the parasites' sugar transporter repertoire (Figure 1). To validate sugar transporter copy number patterns, Pfam, HMM, and BLAST analyses were performed. Within the apicomplexan parasites, the sugar transporter copy number is a variable. It ranges from a high of eight members in the basal-branching taxon *Gregarina niphandrodes* to a low of one member in *Babesia microti* (Figure 1).

The closest known free-living relatives of apicomplexans, *Chromera velia* and *Vitrella brassicaformis*, have more sugar transporters than the apicomplexans at 28 and 24 sugar transporters, respectively (Figure 1). This finding reflects the likely loss of transporters in the parasitic lineages and retention in the free-living relatives. This is consistent with gene loss being a major contributor to reductive genomes in this phylum [10, 31]. The larger number of sugar transporters in chromerids may also mean differential amplifications of gene family members in this lineage.

Sugar transporter copy number varies the most within the Piroplasmida species. *Theileria* species have between three and seven family members while *Babesia* species have only one or two. *Theileria equi* has seven sugar transporter genes, *T. oritentalis* has five, while *T. parva* and *T. annulata* have only three members each. *Babesia bovis* and *B. bigemina* have two members each. *Babesia microti* is the only examined apicomplexan with one sugar transporter. *Babesia microti* has the smallest genome sequence and the smallest number of protein-encoding genes of all apicomplexans examined [32]. The coccidians, *Toxoplasma gondii*, *Neospora caninum*, and *Eimeria tenella* each have five members. *Sarcocystis neurona* is the only examined coccidian with three sugar transporter members. *Cryptosporidium* and *Plasmodium* species have two members each. The variable numbers of gene family members in Piroplasmida species highlight an unusual turnover of sugar transporters in these parasites relative to other apicomplexans. This finding suggests either functional redundancy, expanded sugar transport capabilities, or the possibility that very few sugar transporters are needed to import sufficient host nutrients to sustain their lifestyle in different host niches. Piroplasmida species infect a wide range of vertebrates (primarily mammals and birds) and tick hosts [33–35].

The variable number of sugar transporters observed in apicomplexans especially in Piroplasmida species is not the result of missing annotation. We performed additional analyses (BLAST and HMM searches) of the genome sequences to look for sugar transporters that may not have been annotated (see Section 2.1). Genome sequence assembly, especially missing sequence, may still contribute to the variable number of sugar transporters. To alleviate this effect, we used the latest genome sequence assemblies available. The apicomplexan sugar transporters we identified are found in contiguous regions of sequences (see EuPathDB gene pages), but they have limited synteny to other genera in the phylum [31] so gene loss is difficult to definitively prove. In summary, sugar transporter copy number varies across the Apicomplexa, both within its lineages and with respect to free-living outgroups.

### 3.2. Apicomplexans Have Six Different Phylogenetic Subfamilies of Sugar Transporters

**Figure 2:**
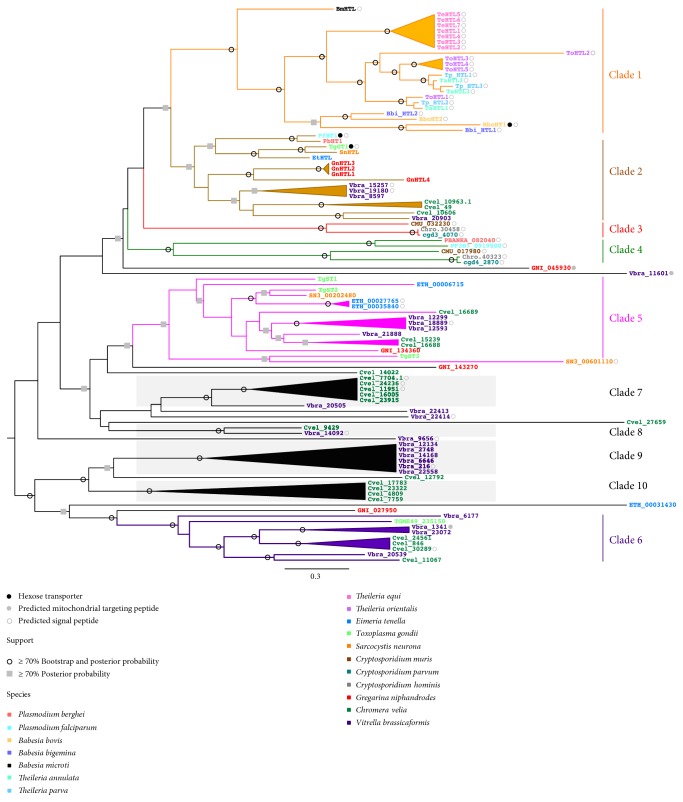
Apicomplexan sugar transporter phylogeny. *Chromera velia* and *Vitrella brassicaformis* sugar transporter gene families were used as outgroups. Phylogenetic clades are colored differentially. Clades outlined with grey boxes are chromerid-specific. Gene identifiers for each species are colored differentially. See Supplementary Table 3 for gene identifiers and aliases. Closed black circle: hexose transporter. Closed grey circle: predicted mitochondrial targeting peptide. Open grey circle: predicted signal peptide. Open black circle: at least 70% bootstrap and posterior probability. Grey square: at least 70% posterior probability.

To identify evolutionary trends in apicomplexan sugar transporter family size and member distribution, we performed phylogenetic analyses. We also analyzed changes in the gene family size using CAFE [23] to look for statistically significant differences. The analyses revealed six major apicomplexan phylogenetic clades or subfamilies (Figures 1 and 2). The free-living chromerid ancestor of apicomplexans contains representatives of at least three of these clades as well as several unique clades not detected in the Apicomplexa (Figure 1). All examined species, except those belonging to *Cryptosporidium,* have sugar transporter family members in clades 1 and 2, henceforth called the pan-apicomplexan subgroup (Figure 1). Some members of this subgroup have been shown experimentally to transport hexoses, indicating a possible phylum-wide conservation of this function [12–14]. The Piroplasmida sugar transporters are restricted to clade 1. However, there is considerable variation in the number of sugar transporters found in each species. The protein sequences of Piroplasmida sugar transporters have predicted signal peptides suggesting they are secreted (Figure 2). The presence of signal peptides may indicate transport roles in the plasma membrane and parasitophorous vacuole (a subcellular compartment that acts as an interface between the parasite and host [36]). According to CAFE, *Theileria equi* and *T. orientalis* have undergone expansions (Figure 1, Supplementary Figure 1). Several *Gregarina niphandrodes* sugar transporters are located within clade 2 and have also undergone expansion; however, unlike the Piroplasmida, family members are also present in other clades. The expansions of sugar transporters in *G. niphandrodes* and Piroplasmida species may indicate specialization of sugar import in these parasites or differing energy requirements. It is known that gene family member variation resulting from differential expansions may be signatures of adaptation to a niche [37] or may suggest emergence of novel biological functions [38]. Expansions may also be due to chance, but this is unlikely in these streamlined genomes.

Clades 3 and 4 consist of sugar transporters found only in *Cryptosporidium* or *Plasmodium*, indicating either their extreme divergence or loss from all the other examined species including the chromerids. The protein sequences of the sugar transporters in clades 3 and 4 have predicted signal peptides suggesting they are secreted (Figure 2). Clades 5 and 6 consist of members from *G. niphandrodes*, coccidians, and chromerids suggesting they may have ancestral sugar import functions, which have subsequently been lost in other examined apicomplexans. Three *T. gondii* sugar transporters are found in clade 5. TgST2 localizes to the tachyzoite plasma membrane. While TgST1 and TgST3 both localize to tachyzoite subcellular compartments and are found to partially colocalize with a dense granule protein [13]. Dense granules are among specialized secretory organelles in the apical complex of apicomplexans that play important roles during infection of host cells by the parasite [39]. We did not find any targeting peptides as part of TgST1 and TgST3. There are four chromerid-specific clades. This observation suggests a loss of several sugar transporter genes that perhaps function as well in apicomplexans. Alternatively, expansion and divergence in extant members of these outgroup species is also a formal possibility.

### 3.3. Divergent Apicomplexan Sugar Transporters Are Ancient Alveolate Genes

#### 3.3.1. Divergent *Plasmodium* Sugar Transporters Are Retained and May Be Sporozoite-Specific

**Figure 3:**
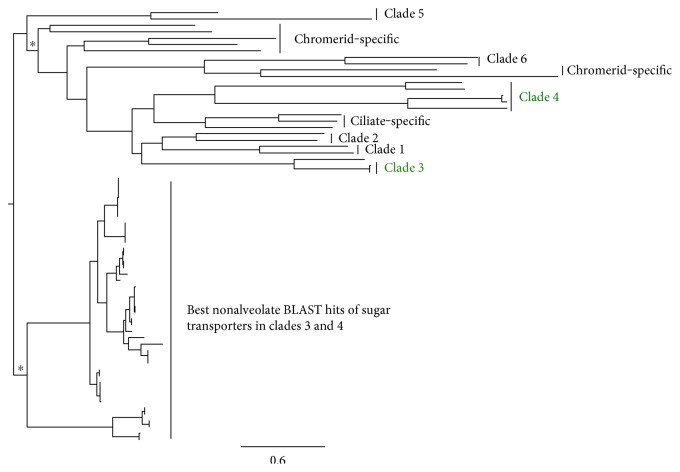
Expanded phylogeny of divergent *Plasmodium* and *Cryptosporidium* as well as representative sugar transporter member from across the tree of life. Representative sugar transporters from across the tree of life are included. Clades containing divergent sugar transporters identified in Figure 2 are colored green. Clade nomenclature according to Figure 1. BLAST hits are for sugar transporters in clades 3 and 4. Asterisk represents at least 95% support.

To determine the origin of divergent sugar transporters present in *Cryptosporidium* and *Plasmodium* species, phylogenetic analyses were expanded to include sugar transporters from diverse organisms across the tree of life (Ciliates, Kinetoplastids, Plants, Red Algae, Amoeba, Opisthokonta, Bacteria, and Archaea). We found that the divergent sugar transporters in *Plasmodium* species (Figure 1: clade 4) cluster with those of ciliates (Figure 3). This implies that the divergent *Plasmodium* transporter-encoding genes are ancestral to alveolates but were lost in most apicomplexans and chromerids. Other possibilities include convergent evolution or phylogenetic artifacts. The divergent *Plasmodium* sugar transporters may represent novel or lineage-specific sugar import functions essential to the lifestyles of the malarial parasites, but this is only a speculation that remains to be confirmed experimentally.

To examine the possible roles of two *Plasmodium* sugar transporters (pan-apicomplexan and divergent), we examined the available protein expression data. Proteomics data from EuPathDB revealed that the pan-apicomplexan conserved *P. falciparum* sugar transporter (PfHT1 – PF3D7_0204700) is expressed in all the life cycle stages examined, while the divergent gene (PF3D7_0919500) is only detected in sporozoites (Table 1). This finding suggests that *P. falciparum* sugar transporters may have a specialized role in malarial parasites. Transcript expression profiles in EuPathDB (data not shown) support sporozoite stage expression of the divergent sugar transporter. The divergent sugar transporter in the rodent malarial parasite *P. yoelii*, PY17X_0823700 (ortholog of PF3D7_0919500), has been localized to the plasma membrane of sporozoites [40], supporting the sporozoite specificity of this divergent group of sugar transporters in *Plasmodium* species (Figure 1: clade 4). While these data do not inform with respect to the function of the divergent protein, there are likely different energy needs in the sporozoite, given the host switch from the mosquito vector to vertebrates in this stage.

#### 3.3.2. Divergent *Cryptosporidium* Sugar Transporters Are Retained

The expanded phylogenetic analyses also revealed that the divergent *Cryptosporidium* sugar transporters (Figure 1: clades 3 and 4) cluster closer to those of alveolates (Figure 3). This finding suggests that the *Cryptosporidium* sugar transporters were retained in this lineage of parasites but were lost in other apicomplexans and chromerids. Other interpretations include convergent evolution or phylogenetic artifacts. It should be noted that the alveolate sugar transporters tend to have longer branches relative to the rest of the tree of life. Therefore, long-branch attraction may play a role in the observed phylogenetic clustering. The divergent *Cryptosporidium* sugar transporters may represent important lineage-specific sugar import functions in these pathogens, but this remains to be proven. *Cryptosporidium* species have different sugars in their oocysts such as hexoses, trehalose, and amylopectin [41, 42]. It may be that the divergent *Cryptosporidium* sugar transporters facilitate import of specific substrate sugars needed by the parasites for these biosynthetic pathways.

## 4. Conclusion

Apicomplexans have at least six distinct phylogenetic subfamilies of sugar transporters. The specific substrates, time, and location of expression of each member of these subfamilies are not known. The little expression and functional data that exist suggest there are spatiotemporal specificities or substrate differences between some sugar transporter family members. There are differences in the protein localization patterns of *T. gondii* and *Plasmodium* sugar transporters [12, 13, 40]. It may also be the case that many of these sugar transporter lineages are redundant in function. Further analyses of apicomplexan sugar transporters, especially those in subgroups that are lacking experimental data, will greatly enhance our understanding of host sugar import capabilities among the parasites.

We demonstrated that sugar transporter copy number variation resulted from expansions in the deep branching apicomplexan, *Gregarina niphandrodes* and Piroplasmida species, combined with differential retentions in some apicomplexan lineages. The gene gain and loss patterns observed here are intriguing and may suggest differing nutritional requirements, but this remains to be proven [38]. Further analyses of sugar transporters in Piroplasmida species and *Gregarina niphandrodes* may reveal insights into specialization of sugar import in these parasites or differing energy requirements. We found that *Cryptosporidium* and *Plasmodium* species have divergent sugar transporters. The divergent *Plasmodium* sugar transporters appear to be restricted to expression only in the sporozoite stage [40], a parasite form that must survive in both vertebrate and mosquito hosts. The divergent *Plasmodium* sugar transporters may reflect adaptations to diverse needs in different host environments.

As additional apicomplexan genome sequences become available, their sugar transporter repertoire should be examined. This will add to our knowledge of sugar transporter evolution and diversity within the Apicomplexa.

## Supplementary Material

Supplementary Figure 1: CAFE estimations of expansions and contractions in the apicomplexan sugar transporter gene family. Supplementary Table 1: Species information and sequence sources. Supplementary Table 2: Parameters for in silico tools. Supplementary Table 3: Sugar transporter gene identifiers and aliases.

## Figures and Tables

**Table 1:** Proteome expression profiles of *Plasmodium falciparum* sugar transporters.

		Blood stage								Mosquito/sexual stage	
Gene ID	Phylogenetic classification	Trophozoite	Schizont	Merozoite	Gametocyte I	Gametocyte II	Gametocyte III	Gametocyte IV	Gametocyte V	Ookinete	Sporozoite
PF3D7_0204700	Clade 2	+	+	+	+	+	nd	nd	+	nd	+
PF3D7_0919500	Clade 4	nd	nd	nd	nd	nd	nd	nd	nd	nd	+

Phylogenetic classification (see Figure 1); +: protein expression is detected; nd: not detected.
