# Supplementary material for: Evolution of the Apicomplexan Sugar Transporter Gene Family Repertoire
Source: Int J Genomics. 2017 May 7;2017:1707231. doi: 10.1155/2017/1707231 (PMC5438862; doi:10.1155/2017/1707231)
Supplement: Supplementary file 1 — Supplementary Figure 1: CAFE estimations of expansions and contractions in the apicomplexan sugar transporter gene family. Supplementary Table 1: Species information and sequence sources. Supplementary Table 2: Parameters for in silico tools. Supplementary Table 3: Sugar transporter gene identifiers and aliases. [file 1707231.f1.pdf]

Supplementary Figures and Tables

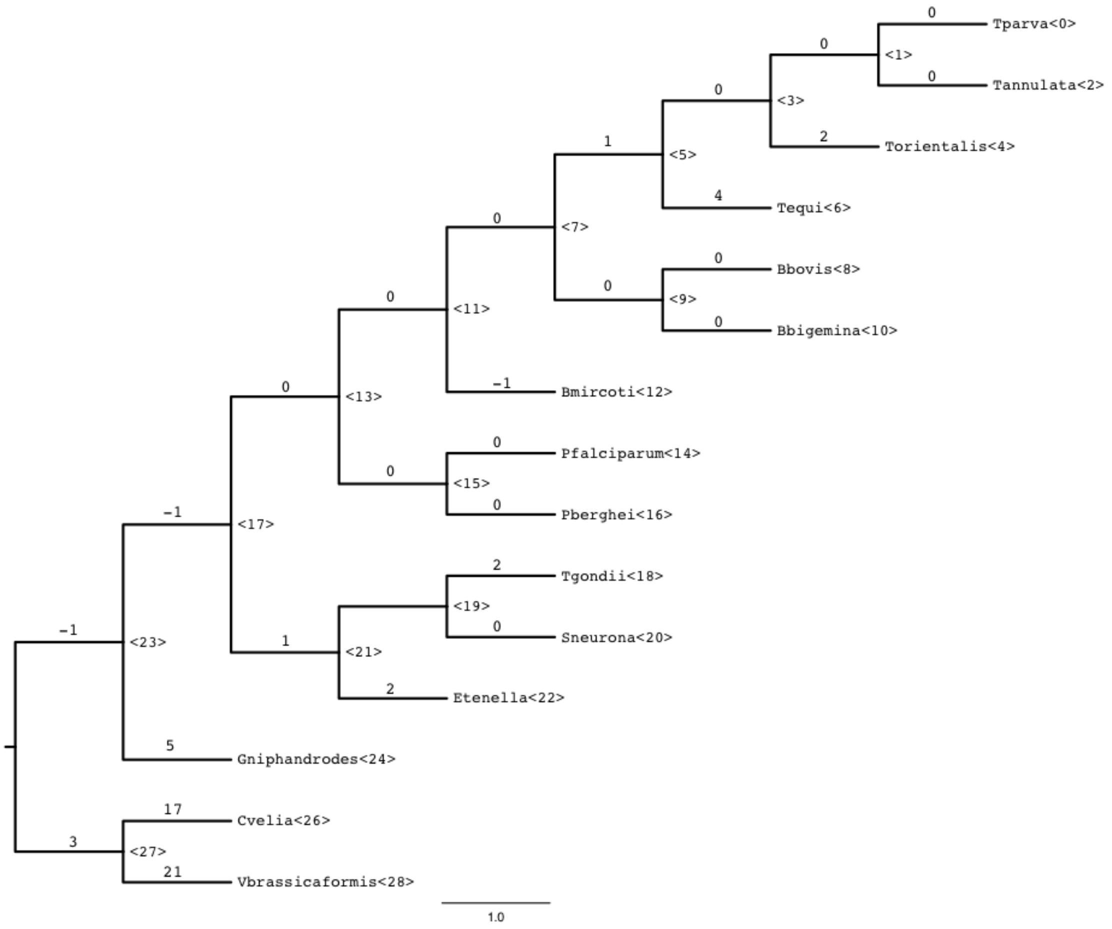

Supplementary Figure 1: CAFE estimations of expansions and contractions in the apicomplexan sugar transporter gene family

The phylogenetic tree shows the mean number of sugar transporters gained or lost per node. Minus indicates a net contraction. Nodes are numbered in angle brackets. The phylogenetic tree was generated by a Bayesian approach using representative sugar transporter protein sequences from clades 1 and 2 (Figure 2).

Supplementary Table 1: Species information and sequence sources

Species	Strain	NCBI Taxon ID	Sequence Source	Database URL	Database Version
Babesia bigemina	BOND	5866	PiroplasmaDB	http://piroplasmadb.org	release 25
Babesia bovis	T2Bo	484906	PiroplasmaDB	http://piroplasmadb.org	release 25
Babesia microti	RI	1133968	PiroplasmaDB	http://piroplasmadb.org	release 25
Theileria annulata	Ankara	353154	PiroplasmaDB	http://piroplasmadb.org	release 25
Theileria equi	WA	1537102	PiroplasmaDB	http://piroplasmadb.org	release 25
Theileria orientalis	Shintoku	869250	PiroplasmaDB	http://piroplasmadb.org	release 25

Theileria parva	Muguga	333668	PiroplasmaDB	http://piroplasmadb.org	release 25
Cryptosporidium hominis	TU502	353151	CryptoDB	http://cryptodb.org	release 25
Cryptosporidium muris	RN66	441375	CryptoDB	http://cryptodb.org	release 25
Cryptosporidium parvum	Iowa II	353152	CryptoDB	http://cryptodb.org	release 25
Gregarina niphandrodes	unknown	110365	CryptoDB	http://cryptodb.org	release 25
Chromera velia	CCMP2878	1169474	CryptoDB	http://cryptodb.org	release 25
Vitrella brassicaformis	CCMP3155	1169540	CryptoDB	http://cryptodb.org	release 25
Eimeria tenella	Houghton	413949	ToxoDB	http://toxodb.org	release 25
Hammondia hammondi	H.H.34	99158	ToxoDB	http://toxodb.org	release 25
Neospora caninum	Liverpool	572307	ToxoDB	http://toxodb.org	release 25
Sarcocystis neurona	SN3	42890	ToxoDB	http://toxodb.org	release 25
Toxoplasma gondii	ME49	508771	ToxoDB	http://toxodb.org	release 25
Plasmodium berghei	ANKA	5823	PlasmoDB	http://plasmodb.org	release 25
Plasmodium falciparum	3D7	36329	PlasmoDB	http://plasmodb.org	release 25
Plasmodium chabaudi	chabaudi	31271	PlasmoDB	http://plasmodb.org	release 25
Plasmodium knowlesi	H	5851	PlasmoDB	http://plasmodb.org	release 25
Plasmodium vivax	Sal-1	126793	PlasmoDB	http://plasmodb.org	release 25
Plasmodium yoelii	17X	352914	PlasmoDB	http://plasmodb.org	release 25

Supplementary Table 2: Parameters for *in silico* tools

**Tool**	**Paramaters**
OrthoMCL	BLAST E-value cutoff: 1e-30
	Inflation: 1.5
	P-value cutoff: 1e-05
	P-ident cutoff: 0
	P-match cutoff: 0
Pfam	e-value cutoff: 1e-05
	pfam DB verson: 27.0
	accession number: PF00083
HMMER	e-value cutoff: 1e-05
tBLASTn	e-value cutoff: 1e-06
	query: identified sugar transporters
	subject: assembled genome sequences of the 22 species
CAFE v3	P-value: 0.050000
	Num of Random: 1000
	Lambda : 1.98162354082669 Mu : 0.73467469847160 & Score: 41.100357
TargetP 1.1	Organism group: Non-plant
	Cutoffs: winner-takes-all (default)
SignalP 4.1	Organism group: Eukaryotes
	D-cutoff values: optimized for correlation

Supplementary Table 3: Sugar transporter gene identifiers and aliases

EupathDB Gene ID	Alias used in Phylogenetic Tree	Organism
cgd4_2870	cgd4_2870	*Cryptosporidium parvum*
cgd3_4070	cgd3_4070	*Cryptosporidium parvum*
Vbra_9656	Vbra_9656	*Vitrella brassicaformis*
Vbra_8597	Vbra_8597	*Vitrella brassicaformis*
Vbra_6646	Vbra_6646	*Vitrella brassicaformis*
Vbra_6177	Vbra_6177	*Vitrella brassicaformis*
Vbra_2748	Vbra_2748	*Vitrella brassicaformis*
Vbra_23072	Vbra_23072	*Vitrella brassicaformis*
Vbra_22558	Vbra_22558	*Vitrella brassicaformis*
Vbra_22414	Vbra_22414	*Vitrella brassicaformis*
Vbra_22413	Vbra_22413	*Vitrella brassicaformis*
Vbra_21888	Vbra_21888	*Vitrella brassicaformis*
Vbra_216	Vbra_216	*Vitrella brassicaformis*
Vbra_20903	Vbra_20903	*Vitrella brassicaformis*
Vbra_20539	Vbra_20539	*Vitrella brassicaformis*
Vbra_20505	Vbra_20505	*Vitrella brassicaformis*
Vbra_19180	Vbra_19180	*Vitrella brassicaformis*
Vbra_18889	Vbra_18889	*Vitrella brassicaformis*
Vbra_15257	Vbra_15257	*Vitrella brassicaformis*
Vbra_14168	Vbra_14168	*Vitrella brassicaformis*
Vbra_14092	Vbra_14092	*Vitrella brassicaformis*
Vbra_1341	Vbra_1341	*Vitrella brassicaformis*
Vbra_12593	Vbra_12593	*Vitrella brassicaformis*
Vbra_12299	Vbra_12299	*Vitrella brassicaformis*
Vbra_12134	Vbra_12134	*Vitrella brassicaformis*
Vbra_11601	Vbra_11601	*Vitrella brassicaformis*
TP03_0064	Tp_HTL3s	*Theileria parva*
TP03_0063	Tp_HTL1	*Theileria parva*
TP01_1069	Tp_HTL2	*Theileria parva*
TOT_030000803	ToHTL4	*Theileria orientalis*
TOT_030000802	ToHTL5	*Theileria orientalis*
TOT_020000891	ToHTL2	*Theileria orientalis*
TOT_010001273	ToHTL3	*Theileria orientalis*
TOT_010001014	ToHTL1	*Theileria orientalis*
TGME49_272500	TgST2	*Toxoplasma gondii*
TGME49_257120	TgST1	*Toxoplasma gondii*
TGME49_235150	TGME49_235150	*Toxoplasma gondii*

TGME49_214320	TgGT1	*Toxoplasma gondii*
TGME49_201260	TgST3	*Toxoplasma gondii*
TA16160	TaHTL1	*Theileria annulata*
TA02485	TaHTL2	*Theileria annulata*
TA02480	TaHTL3	*Theileria annulata*
SN3_00601180	SnHTL	*Sarcocystis neurona*
SN3_00601110	SN3_00601110	*Sarcocystis neurona*
SN3_00202480	SN3_00202480	*Sarcocystis neurona*
PF3D7_0919500	PF3D7_0919500	*Plasmodium falciparum*
PF3D7_0204700	PfHT1	*Plasmodium falciparum*
PBANKA_082040	PBANKA_082040	*Plasmodium berghei*
PBANKA_030250	PbHT1	*Plasmodium berghei*
GNI_143270	GNI_143270	*Gregarina niphandrodes*
GNI_134360	GNI_134360	*Gregarina niphandrodes*
GNI_123980	GnHTL4	*Gregarina niphandrodes*
GNI_060640	GnHTL3	*Gregarina niphandrodes*
GNI_060610	GnHTL2	*Gregarina niphandrodes*
GNI_060510	GnHTL1	*Gregarina niphandrodes*
GNI_045930	GNI_045930	*Gregarina niphandrodes*
GNI_027950	GNI_027950	*Gregarina niphandrodes*
ETH_00035840	ETH_00035840	*Eimeria tenella*
ETH_00031430	ETH_00031430	*Eimeria tenella*
ETH_00027765	ETH_00027765	*Eimeria tenella*
ETH_00021075	EtHTL	*Eimeria tenella*
ETH_00006715	ETH_00006715	*Eimeria tenella*
Cvel_9429	Cvel_9429	*Chromera velia*
Cvel_846	Cvel_846	*Chromera velia*
Cvel_7759	Cvel_7759	*Chromera velia*
Cvel_7704.1	Cvel_7704.1	*Chromera velia*
Cvel_49	Cvel_49	*Chromera velia*
Cvel_4809	Cvel_4809	*Chromera velia*
Cvel_30289	Cvel_30289	*Chromera velia*
Cvel_27659	Cvel_27659	*Chromera velia*
Cvel_24561	Cvel_24561	*Chromera velia*
Cvel_24236	Cvel_24236	*Chromera velia*
Cvel_23915	Cvel_23915	*Chromera velia*
Cvel_23322	Cvel_23322	*Chromera velia*
Cvel_17783	Cvel_17783	*Chromera velia*
Cvel_16689	Cvel_16689	*Chromera velia*

Cvel_16688	Cvel_16688	*Chromera velia*
Cvel_16005	Cvel_16005	*Chromera velia*
Cvel_15239	Cvel_15239	*Chromera velia*
Cvel_14022	Cvel_14022	*Chromera velia*
Cvel_12792	Cvel_12792	*Chromera velia*
Cvel_11951	Cvel_11951	*Chromera velia*
Cvel_11067	Cvel_11067	*Chromera velia*
Cvel_10963.1	Cvel_10963.1	*Chromera velia*
Cvel_10606	Cvel_10606	*Chromera velia*
Chro.40323	Chro.40323	*Cryptosporidium hominis*
Chro.30458	Chro.30458	*Cryptosporidium hominis*
CMU_032230	CMU_032230	*Cryptosporidium muris*
CMU_017980	CMU_017980	*Cryptosporidium muris*
BEWA_049600	TeHTL1	*Theileria equi*
BEWA_048520	TeHTL2	*Theileria equi*
BEWA_047560	TeHTL3	*Theileria equi*
BEWA_045770	TeHTL4	*Theileria equi*
BEWA_043960	TeHTL5	*Theileria equi*
BEWA_028230	TeHTL5	*Theileria equi*
BEWA_025760	TeHTL6	*Theileria equi*
BBOV_IV003180	BboHTL1	*Babesia bovis*
BBOV_IV003170	BboHTL2	*Babesia bovis*
BBM_III01335	BmHTL	*Babesia microti*
BBBOND_0111000	Bbi_HTL2	*Babesia bigemina*
BBBOND_0110990	Bbi_HTL1	*Babesia bigemina*
