# Supplementary material for: Evolution of the Apicomplexan Sugar Transporter Gene Family Repertoire
Source: Int J Genomics. 2017 May 7;2017:1707231. doi: 10.1155/2017/1707231 (PMC5438862; doi:10.1155/2017/1707231)
Supplement: Supplementary file 2 [file 1707231.f2.docx]

**Supplementary Figures and Tables**


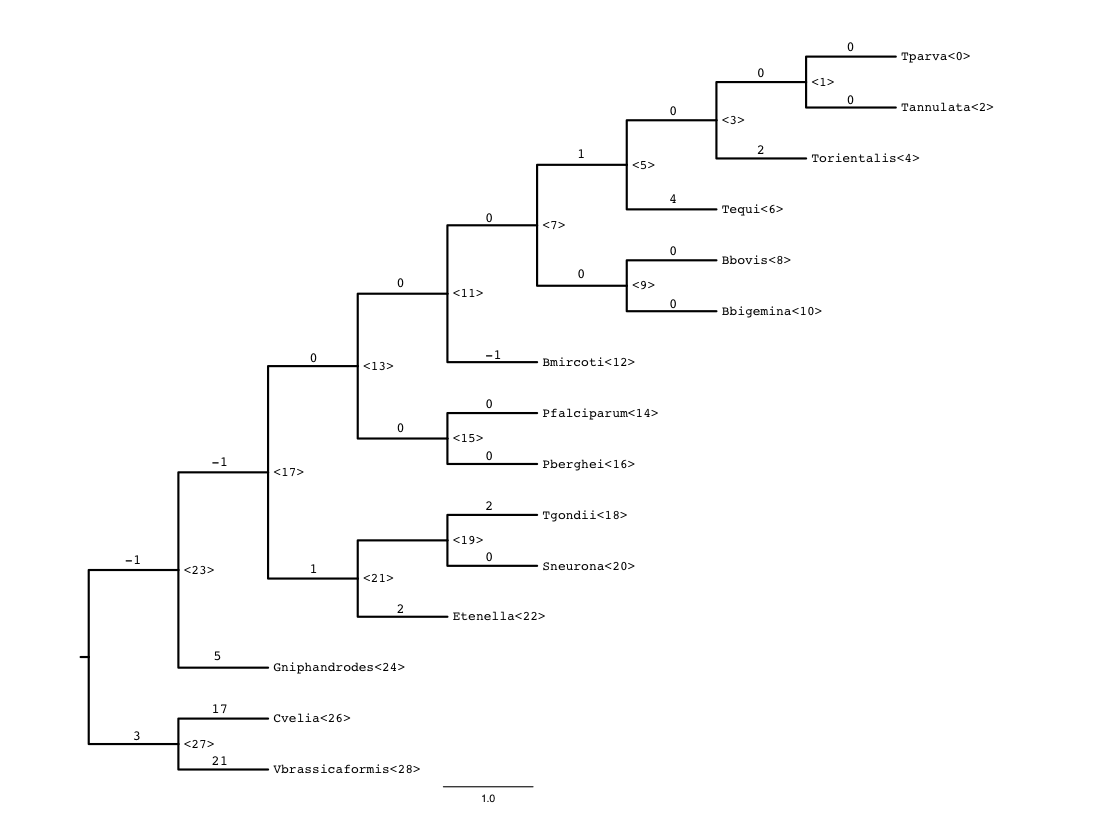


Supplementary Figure 1: CAFE estimations of expansions and contractions in the apicomplexan sugar transporter gene family

The phylogenetic tree shows the mean number of sugar transporters gained or lost per node. Minus indicates a net contraction. Nodes are numbered in angle brackets. The phylogenetic tree was generated by a Bayesian approach using representative sugar transporter protein sequences from clades 1 and 2 (Figure 2).

Supplementary Table 1: Species information and sequence sources

| **Species** | **Strain** | **NCBI Taxon ID** | **Sequence Source** | **Database URL** | **Database Version** |
| --- | --- | --- | --- | --- | --- |
| *Babesia bigemina* | BOND | 5866 | PiroplasmaDB | http://piroplasmadb.org | release 25 |
| *Babesia bovis* | T2Bo | 484906 | PiroplasmaDB | http://piroplasmadb.org | release 25 |
| *Babesia microti* | RI | 1133968 | PiroplasmaDB | http://piroplasmadb.org | release 25 |
| *Theileria annulata* | Ankara | 353154 | PiroplasmaDB | http://piroplasmadb.org | release 25 |
| *Theileria equi* | WA | 1537102 | PiroplasmaDB | http://piroplasmadb.org | release 25 |
| *Theileria orientalis* | Shintoku | 869250 | PiroplasmaDB | http://piroplasmadb.org | release 25 |
| *Theileria parva* | Muguga | 333668 | PiroplasmaDB | http://piroplasmadb.org | release 25 |
| *Cryptosporidium hominis* | TU502 | 353151 | CryptoDB | http://cryptodb.org | release 25 |
| *Cryptosporidium muris* | RN66 | 441375 | CryptoDB | http://cryptodb.org | release 25 |
| *Cryptosporidium parvum* | Iowa II | 353152 | CryptoDB | http://cryptodb.org | release 25 |
| *Gregarina niphandrodes* | unknown | 110365 | CryptoDB | http://cryptodb.org | release 25 |
| *Chromera velia* | CCMP2878 | 1169474 | CryptoDB | http://cryptodb.org | release 25 |
| *Vitrella brassicaformis* | CCMP3155 | 1169540 | CryptoDB | http://cryptodb.org | release 25 |
| *Eimeria tenella* | Houghton | 413949 | ToxoDB | http://toxodb.org | release 25 |
| *Hammondia hammondi* | H.H.34 | 99158 | ToxoDB | http://toxodb.org | release 25 |
| *Neospora caninum* | Liverpool | 572307 | ToxoDB | http://toxodb.org | release 25 |
| *Sarcocystis neurona* | SN3 | 42890 | ToxoDB | http://toxodb.org | release 25 |
| *Toxoplasma gondii* | ME49 | 508771 | ToxoDB | http://toxodb.org | release 25 |
| *Plasmodium berghei* | ANKA | 5823 | PlasmoDB | http://plasmodb.org | release 25 |
| *Plasmodium falciparum* | 3D7 | 36329 | PlasmoDB | http://plasmodb.org | release 25 |
| *Plasmodium chabaudi* | chabaudi | 31271 | PlasmoDB | http://plasmodb.org | release 25 |
| *Plasmodium knowlesi* | H | 5851 | PlasmoDB | http://plasmodb.org | release 25 |
| *Plasmodium vivax* | Sal-1 | 126793 | PlasmoDB | http://plasmodb.org | release 25 |
| *Plasmodium yoelii* | 17X | 352914 | PlasmoDB | http://plasmodb.org | release 25 |

Supplementary Table 2: Parameters for *in silico* tools

| **Tool** | **Paramaters** |
| --- | --- |
| OrthoMCL | BLAST E-value cutoff: 1e-30 |
|  | Inflation: 1.5 |
|  | P-value cutoff: 1e-05 |
|  | P-ident cutoff: 0 |
|  | P-match cutoff: 0 |
|  |  |
| Pfam | e-value cutoff: 1e-05 |
|  | pfam DB verson: 27.0 |
|  | accession number: PF00083 |
|  |  |
| HMMER | e-value cutoff: 1e-05 |
|  |  |
| tBLASTn | e-value cutoff: 1e-06 |
|  | query: identified sugar transporters |
|  | subject: assembled genome sequences of the 22 species |
|  |  |
| CAFE v3 | P-value: 0.050000 |
|  | Num of Random: 1000 |
|  | Lambda : 1.98162354082669 Mu : 0.73467469847160 & Score: 41.100357 |
|  |  |
| TargetP 1.1 | Organism group: Non-plant |
|  | Cutoffs: winner-takes-all (default) |
|  |  |
| SignalP 4.1 | Organism group: Eukaryotes |
|  | D-cutoff values: optimized for correlation |
|  |  |
| BEAST analyses | Number of generations: 10000000 |
|  | Effective Sample Size (Parameters): > 1113 |

Supplementary Table 3: Sugar transporter gene identifiers and aliases

| **EupathDB Gene ID** | **Alias used in Phylogenetic Tree** | **Organism** |
| --- | --- | --- |
| cgd4_2870 | cgd4_2870 | *Cryptosporidium parvum* |
| cgd3_4070 | cgd3_4070 | *Cryptosporidium parvum* |
| Vbra_9656 | Vbra_9656 | *Vitrella brassicaformis* |
| Vbra_8597 | Vbra_8597 | *Vitrella brassicaformis* |
| Vbra_6646 | Vbra_6646 | *Vitrella brassicaformis* |
| Vbra_6177 | Vbra_6177 | *Vitrella brassicaformis* |
| Vbra_2748 | Vbra_2748 | *Vitrella brassicaformis* |
| Vbra_23072 | Vbra_23072 | *Vitrella brassicaformis* |
| Vbra_22558 | Vbra_22558 | *Vitrella brassicaformis* |
| Vbra_22414 | Vbra_22414 | *Vitrella brassicaformis* |
| Vbra_22413 | Vbra_22413 | *Vitrella brassicaformis* |
| Vbra_21888 | Vbra_21888 | *Vitrella brassicaformis* |
| Vbra_216 | Vbra_216 | *Vitrella brassicaformis* |
| Vbra_20903 | Vbra_20903 | *Vitrella brassicaformis* |
| Vbra_20539 | Vbra_20539 | *Vitrella brassicaformis* |
| Vbra_20505 | Vbra_20505 | *Vitrella brassicaformis* |
| Vbra_19180 | Vbra_19180 | *Vitrella brassicaformis* |
| Vbra_18889 | Vbra_18889 | *Vitrella brassicaformis* |
| Vbra_15257 | Vbra_15257 | *Vitrella brassicaformis* |
| Vbra_14168 | Vbra_14168 | *Vitrella brassicaformis* |
| Vbra_14092 | Vbra_14092 | *Vitrella brassicaformis* |
| Vbra_1341 | Vbra_1341 | *Vitrella brassicaformis* |
| Vbra_12593 | Vbra_12593 | *Vitrella brassicaformis* |
| Vbra_12299 | Vbra_12299 | *Vitrella brassicaformis* |
| Vbra_12134 | Vbra_12134 | *Vitrella brassicaformis* |
| Vbra_11601 | Vbra_11601 | *Vitrella brassicaformis* |
| TP03_0064 | Tp_HTL3s | *Theileria parva* |
| TP03_0063 | Tp_HTL1 | *Theileria parva* |
| TP01_1069 | Tp_HTL2 | *Theileria parva* |
| TOT_030000803 | ToHTL4 | *Theileria orientalis* |
| TOT_030000802 | ToHTL5 | *Theileria orientalis* |
| TOT_020000891 | ToHTL2 | *Theileria orientalis* |
| TOT_010001273 | ToHTL3 | *Theileria orientalis* |
| TOT_010001014 | ToHTL1 | *Theileria orientalis* |
| TGME49_272500 | TgST2 | *Toxoplasma gondii* |
| TGME49_257120 | TgST1 | *Toxoplasma gondii* |
| TGME49_235150 | TGME49_235150 | *Toxoplasma gondii* |
| TGME49_214320 | TgGT1 | *Toxoplasma gondii* |
| TGME49_201260 | TgST3 | *Toxoplasma gondii* |
| TA16160 | TaHTL1 | *Theileria annulata* |
| TA02485 | TaHTL2 | *Theileria annulata* |
| TA02480 | TaHTL3 | *Theileria annulata* |
| SN3_00601180 | SnHTL | *Sarcocystis neurona* |
| SN3_00601110 | SN3_00601110 | *Sarcocystis neurona* |
| SN3_00202480 | SN3_00202480 | *Sarcocystis neurona* |
| PF3D7_0919500 | PF3D7_0919500 | *Plasmodium falciparum* |
| PF3D7_0204700 | PfHT1 | *Plasmodium falciparum* |
| PBANKA_082040 | PBANKA_082040 | *Plasmodium berghei* |
| PBANKA_030250 | PbHT1 | *Plasmodium berghei* |
| GNI_143270 | GNI_143270 | *Gregarina niphandrodes* |
| GNI_134360 | GNI_134360 | *Gregarina niphandrodes* |
| GNI_123980 | GnHTL4 | *Gregarina niphandrodes* |
| GNI_060640 | GnHTL3 | *Gregarina niphandrodes* |
| GNI_060610 | GnHTL2 | *Gregarina niphandrodes* |
| GNI_060510 | GnHTL1 | *Gregarina niphandrodes* |
| GNI_045930 | GNI_045930 | *Gregarina niphandrodes* |
| GNI_027950 | GNI_027950 | *Gregarina niphandrodes* |
| ETH_00035840 | ETH_00035840 | *Eimeria tenella* |
| ETH_00031430 | ETH_00031430 | *Eimeria tenella* |
| ETH_00027765 | ETH_00027765 | *Eimeria tenella* |
| ETH_00021075 | EtHTL | *Eimeria tenella* |
| ETH_00006715 | ETH_00006715 | *Eimeria tenella* |
| Cvel_9429 | Cvel_9429 | *Chromera velia* |
| Cvel_846 | Cvel_846 | *Chromera velia* |
| Cvel_7759 | Cvel_7759 | *Chromera velia* |
| Cvel_7704.1 | Cvel_7704.1 | *Chromera velia* |
| Cvel_49 | Cvel_49 | *Chromera velia* |
| Cvel_4809 | Cvel_4809 | *Chromera velia* |
| Cvel_30289 | Cvel_30289 | *Chromera velia* |
| Cvel_27659 | Cvel_27659 | *Chromera velia* |
| Cvel_24561 | Cvel_24561 | *Chromera velia* |
| Cvel_24236 | Cvel_24236 | *Chromera velia* |
| Cvel_23915 | Cvel_23915 | *Chromera velia* |
| Cvel_23322 | Cvel_23322 | *Chromera velia* |
| Cvel_17783 | Cvel_17783 | *Chromera velia* |
| Cvel_16689 | Cvel_16689 | *Chromera velia* |
| Cvel_16688 | Cvel_16688 | *Chromera velia* |
| Cvel_16005 | Cvel_16005 | *Chromera velia* |
| Cvel_15239 | Cvel_15239 | *Chromera velia* |
| Cvel_14022 | Cvel_14022 | *Chromera velia* |
| Cvel_12792 | Cvel_12792 | *Chromera velia* |
| Cvel_11951 | Cvel_11951 | *Chromera velia* |
| Cvel_11067 | Cvel_11067 | *Chromera velia* |
| Cvel_10963.1 | Cvel_10963.1 | *Chromera velia* |
| Cvel_10606 | Cvel_10606 | *Chromera velia* |
| Chro.40323 | Chro.40323 | *Cryptosporidium hominis* |
| Chro.30458 | Chro.30458 | *Cryptosporidium hominis* |
| CMU_032230 | CMU_032230 | *Cryptosporidium muris* |
| CMU_017980 | CMU_017980 | *Cryptosporidium muris* |
| BEWA_049600 | TeHTL1 | *Theileria equi* |
| BEWA_048520 | TeHTL2 | *Theileria equi* |
| BEWA_047560 | TeHTL3 | *Theileria equi* |
| BEWA_045770 | TeHTL4 | *Theileria equi* |
| BEWA_043960 | TeHTL5 | *Theileria equi* |
| BEWA_028230 | TeHTL5 | *Theileria equi* |
| BEWA_025760 | TeHTL6 | *Theileria equi* |
| BBOV_IV003180 | BboHTL1 | *Babesia bovis* |
| BBOV_IV003170 | BboHTL2 | *Babesia bovis* |
| BBM_III01335 | BmHTL | *Babesia microti* |
| BBBOND_0111000 | Bbi_HTL2 | *Babesia bigemina* |
| BBBOND_0110990 | Bbi_HTL1 | *Babesia bigemina* |
